# Supplementary material for: Sex differences in alcohol and tobacco use in Ugandan adults: A log-linear analysis of interaction patterns
Source: PLoS One. 2025 Dec 4;20(12):e0337755. doi: 10.1371/journal.pone.0337755 (PMC12677488; doi:10.1371/journal.pone.0337755)
Supplement: Attachment — Submitted filename: Reviewer Attachment.docx [file pone.0337755.s001.docx]

**Summary Comments**

This manuscript presents a valuable analysis of sex differences in alcohol and tobacco use in Uganda using WHO STEPS 2014 data and log-linear modeling. The statistical approach is appropriate and the results are clearly presented, but several areas require attention before acceptance. First, the introduction needs stronger contextualization with Uganda/East Africa–specific literature. Methodological clarity is needed on sample size after exclusions, definition of “current use,” handling of missing data, and whether survey weights were applied. The unexpected finding of higher alcohol use among women warrants deeper exploration of cultural, socioeconomic, and reporting factors. Limitations should be expanded to acknowledge the dataset’s age, self-report bias, unmeasured confounding, and the use of binary categories. Some references require verification for topical relevance. Inclusion of 95% confidence intervals for prevalence and model parameters, consistent terminology and p-value formatting, and a brief rationale for model selection will improve rigor. Overall, this is a promising paper that merits **major revision** to enhance clarity, contextual relevance, and interpretive depth.

Title & Abstract

- Line 1–19 (Title & Abstract) – Major: The title is informative and reflects the content. The abstract is well-structured, but it omits the sample size, the year of the survey, and a clearer operational definition of “current use.” Including these will improve reproducibility and transparency.
- Line 31–37 – Minor: In reporting prevalence differences, consider adding confidence intervals for key proportions (e.g., 60% vs 38.9%), even in the abstract, for better statistical context.
- Line 36–37 – Minor: When noting non-significant alcohol–tobacco interaction, briefly interpret what this implies in practical terms.

Introduction

- Line 42–74 – Major: The literature review is thorough but is disproportionately weighted toward global and high-income country studies. To strengthen novelty, include more region-specific or Ugandan studies on alcohol/tobacco co-use. This will better situate the findings in local context.
- Line 55–57 – Minor: The narrowing gender gap in alcohol use is mentioned, but the introduction should explain why this is important in Uganda specifically—are similar trends expected or observed locally?
- Line 58–63 – Minor: While log-linear modeling is well introduced, explicitly state why it was chosen over logistic regression for this dataset.

Materials and Methods

- Line 79–88 – Major: Clarify the sample size after excluding missing data, and describe how missingness was handled (e.g., listwise deletion, imputation).
- Line 94–96 – Major: Define “current use” (past 30 days? past year?) for both alcohol and tobacco. This definition is crucial for interpreting prevalence.
- Line 106–110 – Minor: The stratification by sex is stated, but later analysis includes sex interactions—clarify whether stratification was used purely for descriptive purposes or in modeling steps as well.
- Line 117–149 – Minor: The description of models is clear, but the rationale for selecting exactly these five models could be tied to theoretical considerations or previous literature.
- Line 150–168 – Minor: Include a note on whether survey weights from WHO STEPS were applied; if not, acknowledge as a limitation.
- Line 155–167 – Minor: Include information on model diagnostics (e.g., residual analysis) to confirm appropriateness of fit beyond G² and D statistics.

Results

- Line 169–192 – Major: The finding that alcohol use is higher among females is unusual in the context of much of the literature—flag this early and suggest possible reasons (cultural, sampling, reporting bias) before the discussion.
- Table 1 (Line 194) – Minor: Include 95% CIs for all prevalence estimates.
- Line 196–219 – Minor: Provide a clearer transition from fit statistics to the interpretation of interactions, so readers can connect statistical results to substantive meaning.
- Table 2 (Line 221) – Minor: Ensure consistent p-value formatting (e.g., “p < 0.001”).
- Line 223–251 – Major: Table 3’s observed vs expected counts is useful but would benefit from a short interpretation paragraph connecting deviations to specific behavioral patterns.
- Line 254–275 – Minor: In Table 4, include confidence intervals for parameter estimates alongside standard errors.

Discussion

- Line 278–306 – Major: The discussion should provide more insight into why Ugandan women may have higher alcohol use prevalence in this dataset—social changes, economic empowerment, urbanization, or targeted marketing?
- Line 311–312 – Minor: The lack of significant alcohol–tobacco interaction could be explained in greater depth—e.g., does it suggest that these behaviors are influenced by distinct social environments?
- Line 316–317 – Major: Expand on the limitation of using 2014 data—substance use trends may have shifted considerably over a decade.
- Line 317–318 – Minor: The binary categorization of alcohol/tobacco use likely masks important differences in frequency or intensity—acknowledge this limitation explicitly.

Conclusion

- Line 323–356 – Minor: The conclusion is appropriately cautious, but temper policy recommendations to acknowledge the dataset’s age and limitations in variable granularity.

References

- Line 362–448 – Major: Reference #5 (“NTIRE 2025 challenge…”) appears unrelated to the topic and may have been included in error—verify all references for topical relevance.
- Ensure all in-text citations match the reference list and remove any non-cited references.

Figures & Tables

- Figure 1 (Line 193) – Minor: Add confidence intervals or error bars for prevalence bars to convey uncertainty.

Summary Recommendation

Major Revision – Strengthen methodological transparency, contextual interpretation, and reference accuracy. Address the unexpected female-prevalence finding in greater depth and provide fuller discussion of limitations.

**Confidential Comments to the Editor**

This manuscript addresses an important public health topic using a sound and underutilized statistical method (log-linear modeling) applied to nationally representative data. The analysis is competently conducted and clearly presented. The main concern is that the dataset is from 2014 and the discussion does not sufficiently contextualize the findings, especially the unexpected higher prevalence of alcohol use among women, within the Ugandan or East African setting. Methodological details such as sample size post-exclusions, definition of “current use,” and survey weighting are missing and should be addressed before publication. The limitations section also needs expansion to acknowledge self-report bias, unmeasured confounding, and the implications of using binary categories for alcohol/tobacco use. Reference #5 appears unrelated and raises a question of reference accuracy. Overall, I recommend Major Revision. The manuscript has potential for PLOS ONE if the contextualization, interpretation, and methodological transparency are strengthened.
