# Supplementary material for: Sex differences in alcohol and tobacco use in Ugandan adults: A log-linear analysis of interaction patterns
Source: PLoS One. 2025 Dec 4;20(12):e0337755. doi: 10.1371/journal.pone.0337755 (PMC12677488; doi:10.1371/journal.pone.0337755)
Supplement: Attachment — Submitted filename: Response_to_Editors+Reviewer_comments.docx [file pone.0337755.s003.docx]

**Rebuttal to Reviewer and Editor Comments**

**Manuscript title:** Sex Differences in Alcohol and Tobacco Use in Ugandan adults: A Log-Linear Analysis of Interaction Patterns

**Manuscript ID:** PONE-D-25-32619

**Dear Editor,**

We thank you and the reviewers for the thoughtful feedback. Below we respond point-by-point

**Reviewer Comment**

The higher prevalence of alcohol use among females needs further explanation. Please discuss possible scientific reasons beyond this observation.

**Author Response**

We appreciate this insightful comment. We have now expanded the discussion to include potential scientific explanations for the observed higher prevalence of alcohol use among females. Specifically, we note that recent sociocultural shifts in Uganda and similar contexts have led to greater social acceptance of alcohol consumption among women. Targeted marketing strategies toward women, increasing urbanization, and evolving gender roles may also contribute to this trend. Furthermore, psychosocial stressors such as caregiving burdens, economic hardship, and gender-based violence can increase vulnerability to alcohol use as a coping mechanism. Biological factors, including sex differences in alcohol metabolism and sensitivity, may further reinforce these behavioral patterns. These points have been incorporated in the Discussion section

**Reviewer Comment**

Please justify the use of Poisson regression, implemented within the Generalized Linear Model (GLM) framework, in this analysis.

**Author Response**

We thank the reviewer for this valuable comment. We have clarified and justified our use of **Poisson regression within the Generalized Linear Model (GLM) framework** in the revised manuscript. Poisson regression is appropriate in this context because the **cell counts in the contingency table** (derived from combinations of categorical variables: Sex, Alcohol use, and Tobacco use) represent **count data**, which follow a discrete and non-negative distribution.

The **log-linear model** is mathematically equivalent to a **Poisson regression model** with a log link function and no explicit dependent variable, treating all variables symmetrically. This framework allows us to model the natural logarithm of expected counts as a linear function of the categorical predictors and their interactions. Thus, Poisson regression provides an alternative parameterization that enables estimation of coefficients (e.g., main effects and interaction terms), along with their **standard errors, Z-values, and p-values**, facilitating formal statistical inference.

Additionally, the use of the GLM formulation offers flexibility and interpretability advantages—it allows straightforward model comparison (via likelihood ratio tests), extension to nested models, and evaluation of the goodness-of-fit (e.g., using the deviance or G² statistic). This dual approach (loglm and GLM-Poisson) therefore strengthens our analysis by combining model-based inference with descriptive model assessment.

We have now included a brief justification for this methodological choice in the **Statistical Analysis section**

**Reviewer Comment**

Please justify the use of a log-linear model and explain its importance in your study.

**Author Response**

We appreciate the reviewer’s request for clarification. The **log-linear model** was chosen because it provides a robust and symmetric framework for analyzing associations among **categorical variables** without designating any one variable as dependent or independent. In our study, the primary interest was to explore the **interrelationships and interaction effects** among *Sex*, *Alcohol use*, and *Tobacco use*. Unlike logistic regression, which models a single binary outcome, log-linear analysis treats all variables equally and focuses on how the joint distribution of categorical variables deviates from independence.

This makes the log-linear approach ideal for detecting **mutual, joint, and conditional independence structures**, as well as higher-order interactions. It allows us to systematically test whether associations among exposures differ across levels of other factors (e.g., whether the alcohol–tobacco relationship varies by sex). In addition, fitting hierarchical log-linear models enables formal model comparison using **likelihood ratio chi-square (G²)** statistics and **Dissimilarity Indices (D)**, thus ensuring objective model selection.

We have now included this justification and explanation of its importance in the **Statistical Analysis section**

Reviewer Comment

The manuscript omits several potentially important variables that may influence alcohol and tobacco use. Please justify their exclusion and discuss this as a limitation.

Author Response

We appreciate this important observation. We acknowledge that the current analysis did not include certain potentially relevant sociodemographic and behavioral covariates such as age, education, marital status, socioeconomic status, and mental health indicators. These variables were not included because the log-linear modeling framework focuses on examining the structure of associations among categorical variables rather than causal prediction. The study’s primary aim was to evaluate interaction patterns among Sex, Alcohol use, and Tobacco use, using a parsimonious model to assess interdependence among these factors.

Including additional covariates would have substantially increased the dimensionality of the contingency table, leading to sparse cells and unstable parameter estimates—particularly given the categorical nature of the data. To preserve model stability, interpretability, and valid inference, we therefore restricted the model to the three most relevant variables.

We have now clarified this rationale in the Methods section and expanded the Limitations section to explicitly acknowledge that omission of other covariates may limit generalizability and preclude causal interpretation.

**Reviewer Comment**

The manuscript omits several potentially important variables that may influence alcohol and tobacco use. Please justify their exclusion and discuss this as a limitation.

**Author Response**

We appreciate this important observation. We acknowledge that the current analysis did not include certain potentially relevant sociodemographic and behavioral covariates such as age, education, marital status, socioeconomic status, and mental health indicators. These variables were not included because the **log-linear modeling framework** focuses on examining **the structure of associations among categorical variables** rather than causal prediction. The study’s primary aim was to evaluate **interaction patterns** among *Sex*, *Alcohol use*, and *Tobacco use*, using a parsimonious model to assess interdependence among these factors.

Including additional covariates would have substantially increased the dimensionality of the contingency table, leading to sparse cells and unstable parameter estimates—particularly given the categorical nature of the data. To preserve model stability, interpretability, and valid inference, we therefore restricted the model to the three most relevant variables.

We have now clarified this rationale in the **Methods section** and expanded the **Limitations section** to explicitly acknowledge that omission of other covariates may limit generalizability and preclude causal interpretation.

**Reviewer Comment**

How was the sample size derived? The absence of the total sample size from the STEP survey undermines transparency and impedes accuracy. Review the percentage interpretation in Table 1—how is it computed?

**Author Response**

We thank the reviewer for this valuable comment. The sample for this analysis was drawn from the **World Health Organization STEPwise Approach to Noncommunicable Disease Risk Factor Surveillance (STEPS)** conducted in Uganda. The total sample size 3,987 **adults aged 18–69 years]**, representing a nationally representative adult population after applying survey weights and exclusions for missing data.

**Reviewer Comment**

The manuscript should clearly define dependent and independent variables to avoid ambiguity in model specification.

**Author Response**

We appreciate this valuable clarification. In log-linear modelling, all categorical variables are treated symmetrically—there is no formal distinction between dependent and independent variables. The purpose of this method is to evaluate **patterns of association and interaction** among categorical variables rather than to predict one variable from others.

In our analysis, *Sex* was included as a **stratification (confounding) variable**, not as an outcome. Alcohol and tobacco use were modeled jointly with sex to assess whether their co-occurrence varied across sex strata. We have now explicitly clarified this conceptual framework and terminology in the **Methods section** to prevent ambiguity in the model specification.

This study is a secondary data analysis of the 2014 Uganda WHO STEPwise approach to Surveillance (STEPS) survey, a nationally representative, cross-sectional survey collecting data on Non-Communicable disease risk factors among adults aged 18–69 years.

The STEPS survey of non-communicable disease (NCD) risk factors in Uganda was carried out from April, 2014 through June, 2014. Uganda carried out Step 1, Step 2 and Step 3. Socio demographic and behavioral information was collected in Step 1. Physical measurements such as height, weight and blood pressure were collected in Step 2. Biochemical measurements were collected to assess blood glucose and cholesterol levels in Step 3. The survey was a population-based survey of adults aged 18 69. A Multistage sample design was used to produce representative data for that age range in Uganda. A total of 3987 adults participated in the survey. The response rate was 99.0% for STEPs 1 & 2 and 92.2% for all STEPs 1,2 and 3. A repeat survey was planned for 2024 but funds did not permit.

**Reviewer Comment**

Elaborate WHO-STEP Survey for Readers.

**Author Response**

We appreciate this important suggestion. We have now elaborated on the **World Health Organization STEPwise Approach to Noncommunicable Disease Risk Factor Surveillance (WHO STEPS)** survey in the *Methods* section to provide readers with sufficient background on its purpose, methodology, and relevance.

**Reviewer Comment**

Can the authors provide information on the locations where this data was collected? Was it a mixture of urban and rural?

**Author Response**

We thank the reviewer for this important observation. We have now clarified the sampling framework of the WHO STEPS survey to specify that data were collected from both **urban and rural areas across Uganda**, ensuring national representativeness. The survey design explicitly accounts for regional, urban–rural, and demographic diversity, which enhances the generalizability of the findings.

**Reviewer Comment**

Line 106–110 – Minor: The stratification by sex is stated, but later analysis includes sex interactions—clarify whether stratification was used purely for descriptive purposes or in modelling steps as well.

**Author Response**

We thank the reviewer for this helpful observation. We have clarified that **sex was used both as a stratification variable in descriptive analyses and as an interaction factor in the log-linear modelling phase**. In the descriptive stage, stratification by sex allowed comparison of prevalence rates across males and females. In the modelling stage, inclusion of sex interactions (Sex × Alcohol and Sex × Tobacco) enabled assessment of whether associations between alcohol and tobacco use differed by sex. We have revised the *Methods* section accordingly to make this distinction explicit.

**Reviewer Comment**

The lack of significant alcohol–tobacco interaction could be explained in greater depth—e.g., does it suggest that these behaviours are influenced by distinct social environments?

**Author Response**

We thank the reviewer for this valuable observation. In response, we have expanded the discussion to interpret the non-significant alcohol–tobacco interaction in greater behavioral and sociocultural context. The added paragraph clarifies that the absence of a strong co-use effect likely reflects the influence of different social, cultural, and situational factors that shape alcohol and tobacco use independently in Uganda, rather than shared behavioral environments.

**Reviewer Comment**

Expand on the limitation of using 2014 data—substance use trends may have shifted considerably over a decade.

**Author Response**

We appreciate this insightful comment. We have now expanded the *Limitations* section to explicitly acknowledge the temporal gap between data collection (2014) and the present, emphasizing how evolving cultural, economic, and policy contexts may have influenced alcohol and tobacco use patterns since then. This clarification improves the transparency of the study’s temporal relevance and guides interpretation within the proper historical context.

**Reviewer Comment**

Is that a statistical modeling study or?

**Author Response**

We appreciate the reviewer’s request for clarification. This study is indeed a **statistical modeling study** employing **log-linear modeling** within the **Generalized Linear Model (GLM)** framework. The analysis was designed to evaluate the **joint and conditional associations** between categorical variables—specifically sex, alcohol use, and tobacco use—rather than to estimate causal effects. Accordingly, all variables were treated as **response variables**, and the model assessed how their interrelationships jointly explained the observed frequency distribution. This approach aligns with the classical use of log-linear analysis in contingency table modeling to explore associations and interaction structures among categorical factors.
